# Rate Versus Rhythm Control in a Multimorbid Patient With Tachy–Brady Syndrome: Evaluation of Clinical Outcomes and Pacing Dependency

**DOI:** 10.1002/ccr3.72809

**Published:** 2026-06-14

**Authors:** Reem Fakak, Emma Nordahl, Joseph Moutiris

**Affiliations:** ^1^ Department of Basic and Clinical Sciences University of Nicosia Medical School Nicosia Cyprus; ^2^ Department of Cardiology Evangelismos Hospital Paphos Cyprus

**Keywords:** atrial fibrillation, multimorbidity, pacemaker, rate control, rhythm control, tachy–brady syndrome

## Abstract

This case report describes a 77‐year‐old man with multiple comorbidities, including atrial fibrillation, heart failure with mildly reduced ejection fraction, hypertension, diabetes mellitus, and prior coronary artery bypass grafting, who presented with severe hypertension and dyspnoea. The patient was referred for physiologic pacemaker implantation to enable safe and ongoing rate control. Persistent tachy–brady syndrome complicated both rate and rhythm control strategies, ultimately necessitating evaluation for pacemaker implantation. This case highlights the clinical dilemma between rate and rhythm control in multimorbid patients with tachy–brady syndrome and demonstrates how management choices influence pacing dependency and the clinical outcomes.

## Key Clinical Message

In multimorbid patients with atrial fibrillation, tachy–brady syndrome complicates both rate and rhythm control, as therapies may provoke symptomatic bradycardia or be poorly tolerated by patients. Early recognition of the need for permanent cardiac pacing is therefore crucial.

## 1 Introduction

Atrial fibrillation (AF) is the most common type of sustained arrhythmia worldwide and is particularly associated with advancing age and multimorbidity. Comorbid conditions, particularly heart failure (HF), hypertension, and structural heart disease, contribute to AF progression and adverse outcomes [1]. In 2019, 59 million individuals were estimated to be living with AF, with projections indicating a continued rise [2, 3]. Contemporary American College of Cardiology (ACC), American Heart Association (AHA), American College of Chest Physicians (ACCP), Heart Rhythm Society (HRS), and European Society of Cardiology (ESC) guidelines from 2023 and 2024 emphasize early, individualized management to limit disease progression, hospitalisations, and mortality.

Tachy–brady syndrome (TBS), a subtype of sinus node dysfunction, is characterized by alternative bradyarrhythmia and supraventricular tachyarrhythmias, most commonly AF. In multimorbid patients, TBS complicates AF management by increasing symptomatic pauses and limiting tolerance to both rate‐ and rhythm‐control therapy [4, 5, 6]. These challenges necessitate careful consideration of catheter ablation and pacemaker implantation.

In practice, the choice between rhythm and rate‐control strategies in TBS must consider the degree of atrial structural disease, comorbidity burden, and patient tolerance of medical therapy. Patients with early‐stage AF and limited atrial remodeling may be suitable candidates for rhythm‐control approaches, whereas those with advanced structural changes or significant bradyarrhythmia may require pacing support to enable safe rate‐control [4, 5, 6, 7, 8]. Early recognition of bradycardia that limits medication use is crucial, as timely pacemaker implantation can reduce hospitalizations and prevent refractory treatment [5].

Despite these developments, prospective data guiding the optimal sequencing of ablation and pacing strategies in multimorbid TBS patients remain limited [5]. This case illustrates the practical limitations of guideline‐directed rate and rhythm control strategies when sinus node dysfunction and multimorbidity coexist, further highlighting how TBS influences the balance between rate‐ and rhythm‐control and how these decisions affect pacing‐dependency.

## 2 Case History and Examination

A 77‐year‐old man presented with severe hypertension and mild dyspnoea. His past medical history included AF, coronary artery disease (CAD), heart failure with mildly reduced ejection fraction (HFmrEF), New York Heart Association Class III, diabetes type II, and long‐standing hypertension. The patient had also undergone prior coronary artery bypass grafting (CABG), consisting of a left internal mammary artery (LIMA) graft to the left anterior descending (LAD) artery and a saphenous vein graft (SVG) to the right coronary artery (RCA). Cardiovascular examination revealed a heart rate of 70 beats per minute (bpm), blood pressure of 183/67 mmHg, oxygen saturation of 90% on room air, a systolic regurgitant murmur at the apex, reduced breath sounds with bibasal crackles, and lower‐limb oedema.

### 2.1 Differential Diagnosis

Differential diagnosis included acute decompensated heart failure precipitated by severe hypertension and atrial fibrillation, hypertensive emergency with pulmonary congestion, and tachy–brady syndrome secondary to sick sinus dysfunction in the setting of atrial fibrillation.

### 2.2 Investigations

**FIGURE 1:**
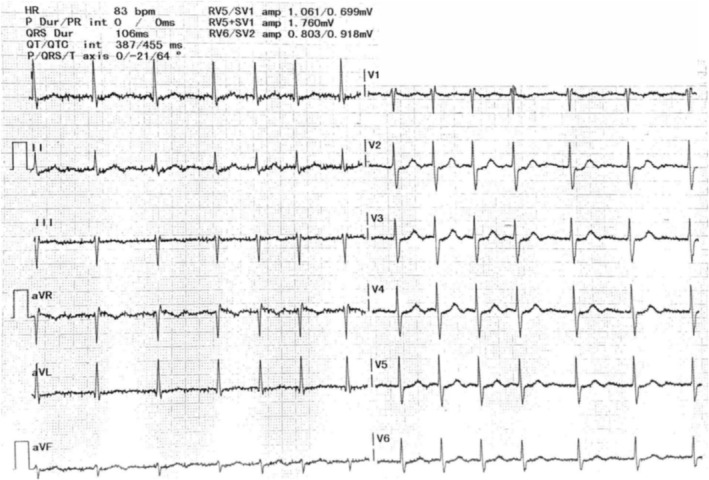
Electrocardiogram confirming atrial fibrillation.

**FIGURE 2:**
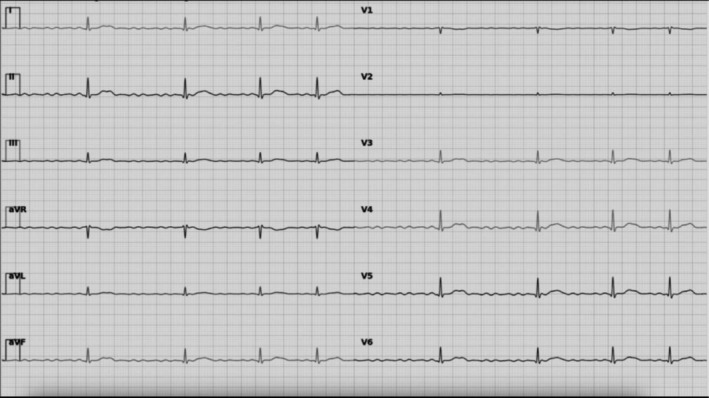
Atrial fibrillation with slow ventricular response.

**FIGURE 3:**
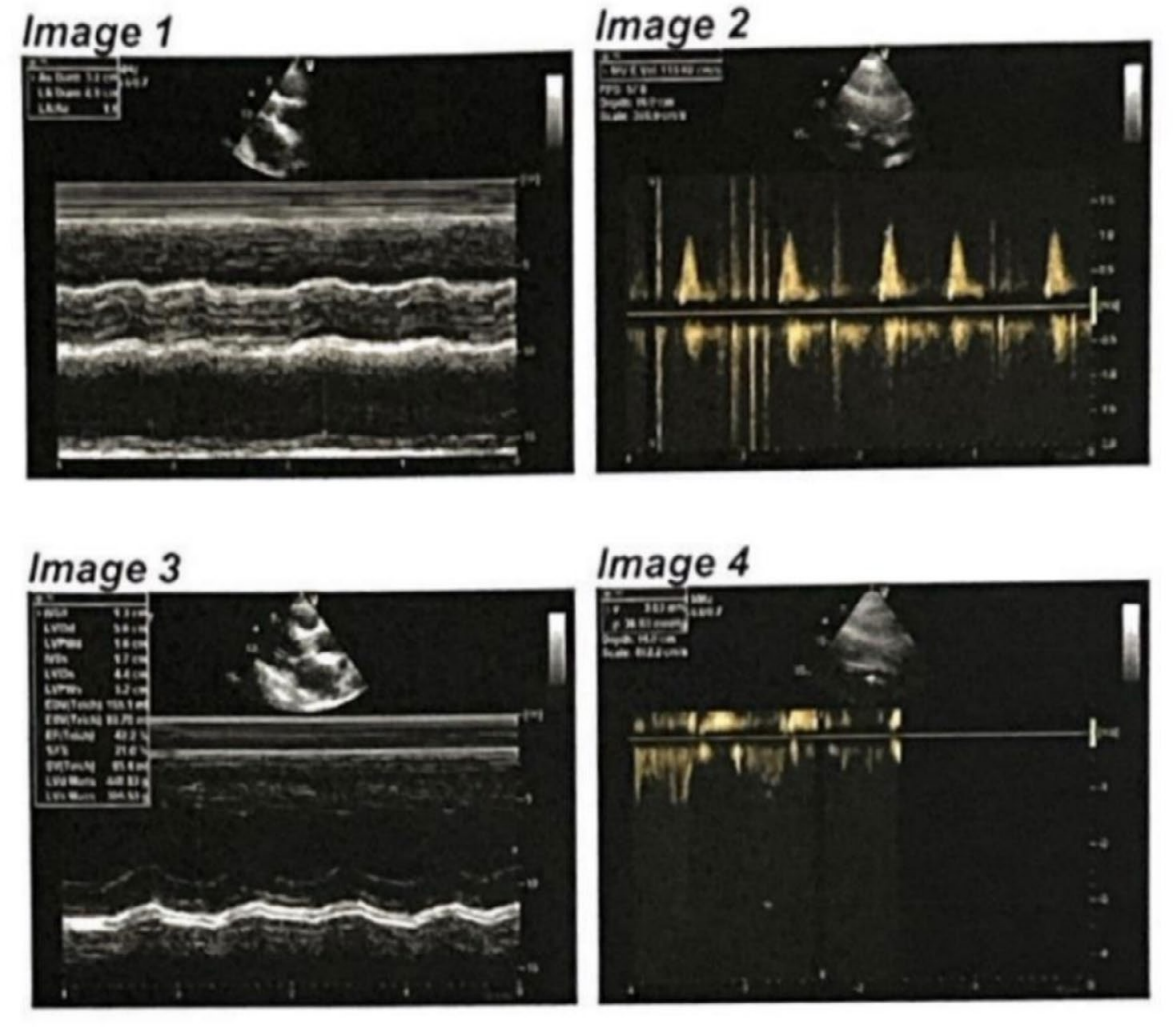
Echocardiogram performed upon hospitalization shows a moderately dilated left atrium (4.9 cm), moderately impaired left ventricular systolic function (EF = 42.4%), and an elevated right ventricular systolic pressure (40–45 mmHg).

**FIGURE 4:**
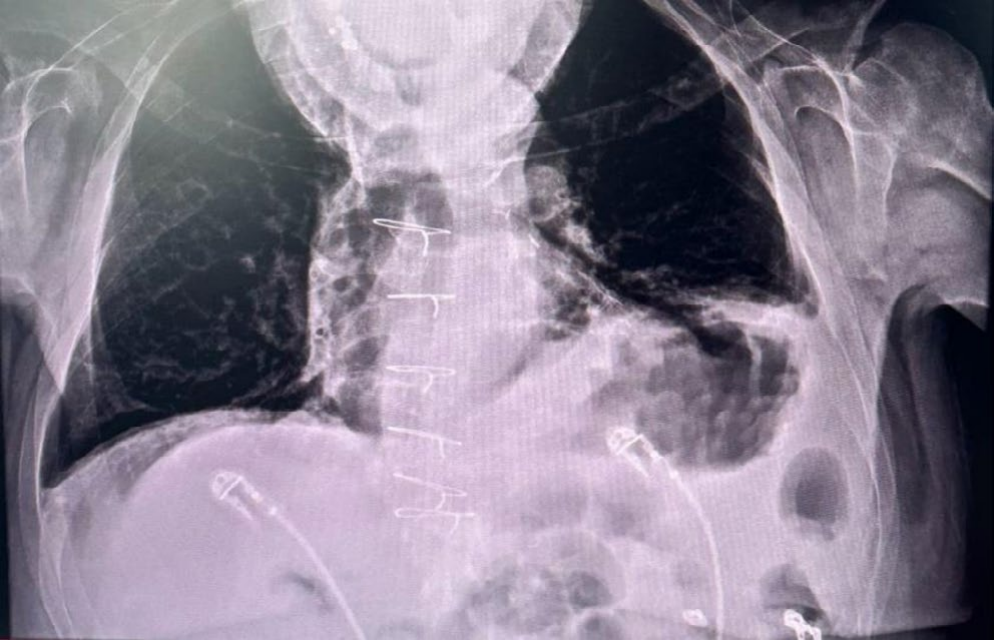
Chest X‐ray posterior–anterior (PA) showing left‐sided heart failure and slight atelectasis.

Initial electrocardiogram (ECG) revealed normofrequent AF [Figure 1]. Four days later, the patient reverted to sinus rhythm without pharmacological or electrical cardioversion. The patient subsequently converted back to AF within 6 h [Figure 2]. Echocardiography revealed a mildly dilated left atrium (LA) measuring 4.9 cm, with a moderately reduced left ventricular ejection fraction of 40%–45% (LVEF). Additional findings included mild aortic, mild‐to‐moderate mitral, and tricuspid regurgitation and a right ventricular systolic pressure (RVSP) of 40–45 mmHg [Figure 3]. Chest radiography (CXR) findings were consistent with left‐sided heart failure and mild atelectasis [Figure 4].

Laboratory evaluation revealed abnormalities relevant to both his cardiovascular status and underlying comorbidities. Biochemistry demonstrated mild chronic macrocytic anemia (hemoglobin 11.2–12 g/dL; mean elevated mean corpuscular volume (MCV) approximately 120 fL); however, folate levels were not tested. There was no history of alcohol consumption. The anemia remained stable during hospitalization and was managed conservatively. NT‐proBNP was markedly elevated on admission, peaking at 2900 ng/L before decreasing to 874 ng/L after treatment (normal value < 450 pg/mL). The cardiac troponin levels were within normal limits. Renal function declined modestly during hospitalization, with an estimated glomerular filtration rate (eGFR) of 46.8 mL/min/1.73 m^2^. Coagulation studies demonstrated prolonged prothrombin time and elevated international normalized ratio, although the patient was not on warfarin. The liver function tests were normal.

### 2.3 Management and Treatment Course

Prior to admission, the patient's regular medications included apixaban 5 mg, pentoxifylline 400 mg twice daily, empagliflozin 10 mg, and allopurinol 300 mg once daily. Upon presentation, antihypertensive therapy was initiated with intravenous isosorbide mononitrate (ISMN) administered as an infusion, followed by transition to oral ISMN (20 mg twice daily) once blood pressure was stabilized. In addition, bisoprolol 2.5 mg, ramipril 5 mg once daily, and furosemide 40 mg twice daily were added. However, rhythm instability persisted, prompting the evaluation for pacemaker implantation.

### 2.4 Tachy–Brady Characteristics

Telemetry monitoring revealed alternating periods of tachycardia and bradycardia, with heart rates dropping to 40–45 bpm and alternating with a rapid heart rate of up to 140 bpm. These fluctuations were associated with symptoms. Bradycardia occurred both during sinus rhythm and during transitions out of AF, suggestive of tachy–brady syndrome.

## 3 Discussion

### 3.1 Rate Versus Rhythm Control in Tachy–Brady Syndrome

TBS complicates AF in multimorbid frail adults. Rate control, typically with beta‐blockers and/or digoxin in the context of HF, although beneficial, may, however, exacerbate bradycardia and increase the likelihood of pacing requirements [4, 9, 10]. Conversely, rhythm control, especially via catheter ablation, may reduce AF burden and occasionally eliminate the need for pacing, although in patients with sinus node dysfunction, rhythm‐control strategies are associated with a high recurrence rate of approximately 50% in persistent AF and 10%–40% in paroxysmal AF [7, 11, 12].

### 3.2 Impact of Multimorbidity

The options for antiarrhythmic therapy are limited due to co‐morbidities such as HF. Class IC antiarrhythmics are contraindicated in HF, and amiodarone has significant side effects, particularly among elderly multimorbid and frail patients. In the present case, amiodarone was not initiated due to the risk of exacerbating bradycardia when combined with beta‐blocker therapy. Thus, catheter ablation should be considered for rhythm control. However, suitability is reduced by age, atrial enlargement and comorbidities [5, 13, 14]. Therefore, catheter ablation was not pursued due to advanced age, multimorbidity, and coexisting sinus node dysfunction, which reduced the likelihood of sustained benefit.

### 3.3 Pacing Considerations

In tachy–brady syndrome, pacing may be required to achieve adequate rate‐control [15]. When pacing is required in patients with HF, physiologic pacing strategies like cardiac resynchronisation therapy (CRT) and His‐bundle pacing are preferred over conventional right ventricular pacing to preserve left ventricular (LV) function and improve symptoms [16, 17, 18]. Additionally there is a reduced risk of pacing‐induced cardiomyopathy with physiologic modalities over conventional RV pacing [18].

Recurrent bradycardia while on essential rate‐control therapy made pacemaker implantation appropriate for the patient in this case. Physiologic LV pacing approaches may preserve LV function effectively while facilitating adequate pharmacological rate control and alleviating the patient's symptoms [10, 18]. Given that the patient's EF was above 35% and the QRS complex was less than 120 ms, CRT was not indicated, and a His‐bundle pacing approach may preserve LV function effectively.

## 4 Conclusion

This case illustrates how tachy–brady syndrome in multimorbid, frail AF patients complicate the decision between rate and rhythm control therapy. Multimorbidity restricts rhythm‐control options, whereas rate‐control therapy increases the risk of symptomatic bradycardia, often necessitating permanent pacing. Understanding how these management choices influence pacing dependency is crucial for tailoring therapy for patients with complex AF.

## Author Contributions

**Reem Fakak:** conceptualization, writing – original draft. **Emma Nordahl:** conceptualization, writing – original draft. **Joseph Moutiris:** resources, writing – review and editing.

## Funding

The authors have nothing to report.

## Consent

Written informed consent was obtained from the patient for publication of this case report and associated images.

## Conflicts of Interest

The authors declare no conflicts of interest.

## Data Availability

The authors have nothing to report.
